# The impact of exercise on the gut microbiota in middle-aged amateur serious runners: a comparative study

**DOI:** 10.3389/fphys.2024.1343219

**Published:** 2024-04-26

**Authors:** Rui Duan, Yu Liu, Yonglian Zhang, Jinrong Shi, Rong Xue, Ruijie Liu, Yuanxin Miao, Xianfeng Zhou, Yongling Lv, Hexiao Shen, Xiongwei Xie, Xu Ai

**Affiliations:** ^1^ Jingmen Central Hospital, Hubei Clinical Medical Research Center for Functional Colorectal Diseases, Jingmen, Hubei, China; ^2^ Research Institute of Agricultural Biotechnology, Jingchu University of Technology, Jingmen, Hubei, China; ^3^ School of Life Sciences and Health Engineering, Hubei University of Technology, Wuhan, China; ^4^ Maintainbiotech Ltd., Wuhan, Hubei, China; ^5^ College of Life Science and Technology, Huazhong University of Science and Technology, Wuhan, China

**Keywords:** serious runners, gut microbiota, exercise, microbial diversity, core genera

## Abstract

**Introduction:**

Exercise, health, and the gut microbiota (GM) are strongly correlated. Research indicates that professional athletes, especially ultra-marathon runners, have unique GM characteristics. However, more research has focused on elite athletes, with little attention given to amateur sports enthusiasts, especially those in the middle-aged population. Therefore, this study focuses on the impact of long-term running on the composition and potential functions of the GM in middle-aged individuals.

**Methods:**

We compared the GM of 25 middle-aged serious runnerswith 22 sedentary healthy controls who had minimal exercise habitsusing 16S rRNA gene sequencing. Additionally, we assessed dietary habits using a food frequency questionnaire.

**Results and Discussion:**

Statistical analysis indicates that there is no significant difference in dietary patterns between the control group and serious runners. Diversity analysis results indicate that there is no significant difference in α diversity between the two groups of GM, but there is a significant difference in β diversity. Analysis of the composition of GM reveals that *Ruminococcus* and *Coprococcus* are significantly enriched in serious runners, whereas *Bacteroides, Lachnoclostridium*, and *Lachnospira* are enriched in the control group. Differential analysis of functional pathway prediction results reveals significant differences in the functional metabolism levels of GM between serious runners and the control group. Further correlation analysis results indicate that this difference may be closely related to variations in GM. In conclusion, our results suggest that long-term exercise can lead to changes in the composition of the GM. These changes have the potential to impact the overall health of the individual by influencing metabolic regulation.

## 1 Introduction

The human gut harbors over 100 trillion microorganisms, and the characteristics of the GM vary among individuals (Thursby and Juge, 2017). Over the past decade, GM has been extensively studied, encompassing nearly all areas of the life sciences (Khatoon et al., 2023; Su et al., 2023; Wang et al., 2023). Human GM is a unique ecosystem that is acquired at birth through maternal inheritance and is influenced by factors such as diet and the environment. As individuals age, the composition and function of the GM gradually change (Brooks et al., 2023; Santaella-Pascual et al., 2023; Thriene and Michels, 2023). GM includes bacteria, fungi, viruses, and protozoa, which collectively perform a series of processes including digestion, absorption, metabolism, and other related functions to maintain the health of the host (Sokol, 2019; Fujisaka et al., 2023). Research has shown that there is an imbalance in the GM of patients with various metabolic disorders, immune system disorders, and cardiovascular and cerebrovascular diseases (Milano et al., 2022; Chu et al., 2023; Lupu et al., 2023; Upadhyay Banskota et al., 2023). Interventions based on the GM, such as antibiotics, fecal microbiota transplantation (FMT), and probiotic supplementation, have also shown significant benefits in the treatment of various diseases, particularly those affecting the digestive system, such as gastrointestinal disorders (Bajaj et al., 2021; Xu et al., 2021; Zhong et al., 2021; Kumari et al., 2022). In addition, scientific exercise interventions can modify the composition of the GM, thereby contributing to overall physical and mental wellbeing.

As is well known, physical exercise can help maintain good health by promoting metabolism, and sports enthusiasts often have a stronger immune system (Caspersen et al., 1985; Ortega et al., 2008; Karhu et al., 2017). Recent studies suggest that moderate physical exercise may benefit the diversity of the GM and increase the presence of beneficial microorganisms. In addition, exercise can enhance the synthesis of short-chain fatty acids and improve carbohydrate metabolism (Rankin et al., 2017; Zhao et al., 2018; Dziewiecka et al., 2022; Kulecka et al., 2023). A recent study found that young people who lack exercise have higher levels of pro-inflammatory gut bacteria and lower immune resistance than elite athletes and physically active individuals (Xu et al., 2022). Additionally, many nutritionists are paying attention to the impact of diet on the GM of athletes. High-protein diet may have a detrimental effect on the GM, reducing the population of beneficial bacteria responsible for producing short-chain fatty acids (Jang et al., 2019; Lee et al., 2019; Huang et al., 2020; Lin et al., 2020). However, probiotic supplementation can improve muscle strength and enhance exercise endurance. Although an increasing number of studies are beginning to focus on the potential link between exercise, GM, and health, most of the research seems to be centered on a small subset of specific populations, such as elite athletes and ultra-endurance marathon runners (Zhao et al., 2018; Grosicki et al., 2019; Xu et al., 2022). There is little mention of nonprofessional sports enthusiasts, especially among middle-aged and elderly populations. Further research in this area is required.

In the 21st century, with an increasingly aging population, health issues of middle-aged and elderly people cannot be ignored. Among them, a group of people choose to focus on running as a cost-effective form of exercise during their free time. They are either drawn to this activity or motivated by the desire for improved health and happiness. They invest a significant amount of time in regular training, but they do not adhere to the strict training schedules and dietary patterns of professional athletes. Pedersen et al. refer to individuals who run at least 40 km per week and maintain or improve their performance as “serious runners” (Pedersen et al., 2018). Nevertheless, compared with elite athletes, they still fall into the category of amateur sports enthusiasts. In short, this group fell between elite athletes and casual sports enthusiasts. Compared to the high standards required to become an elite athlete, joining and becoming a “serious runner” is relatively easy for most ordinary people. Therefore, it is important to explore the characteristics of the GM in this “reachable” behavioral group.

To compare the GM composition of middle-aged serious runners with that of sedentary middle-aged individuals, a group of healthy middle-aged volunteers was selected from the Jingmen Long Distance Running Association. We compared their fecal 16S rRNA sequencing results with those of a healthy control group, and further analyzed potential functional differences. The findings of this study have significant implications for exercise and health in middle-aged and elderly populations.

## 2 Materials and methods

### 2.1 Participants

Running volunteers for this study were recruited from the Jingmen Long-Distance Running Association. Basic information, including age, gender, height, weight, resting heart rate, average weekly running distance (Km/week, recorded by KEEP APP), other lifestyle habits, and medical history, was collected through questionnaires. A survey on daily dietary habits was conducted, taking into account a previous study by Liang et al. (2019). The inclusion criteria were as follows:

(1) Age range: 40–60 years old.
(2) The average weekly running distance exceeded 40 km, and the duration of adherence was more than 1 year.
(3) There was no hospitalization or need for parenteral nutrition or antibiotic treatment in the last 6 months.
(4) Have not consumed excessive alcohol (≤15 g/per day) or psychoactive substances in the last 6 months.
(5) Have not participated in a clinical trial in the last 6 months.
(6) There were no cardiovascular, immune, gastrointestinal, or metabolic diseases.
(7) Without specific dietary preferences.

A total of 25 serious runners who met rigorous criteria were recruited. 22 healthy individuals with little or no exercise habits were selected from the medical examination department as the control group. They did not have a history of suspected gastrointestinal symptoms, cardiovascular disease, immune system disease, gastrointestinal disease, metabolic system disease, or any medication use in the last 6 months. They provided the exercise data for the most recent month by recall.

All subjects volunteered to participate in the study and provided written informed consent. The study complied with the principles of the Declaration of Helsinki and was approved by the Ethics Committee of Jingmen Central Hospital.

### 2.2 Sample collection

All participants collected fecal samples at home using sterile fecal collection tubes, which consisted of a polystyrene plastic tube and a lid with a sampling spoon. The samples were then transported to the laboratory using dry ice storage. Each sample was divided into two 1.5 mL Eppendorf tubes and stored at −80°C until DNA extraction. It is important to note that in order to avoid potential fluctuations in the microbial composition of fecal samples before and after running, we requested all participants to collect their morning fecal samples. This is because running typically occurs in the afternoon or evening. Interestingly, the majority of them maintained a habit of defecating in the morning, while a very small number of individual participants did not show a regular pattern in their defecation habits.

### 2.3 Amplification and high-throughput sequencing of 16S rRNA gene

After collecting all the samples, DNA extraction was performed using the HiPure Stool DNA Mini Kit (Magen, Guangzhou, China), following the manufacturer’s instructions. Subsequently, PCR amplification targeting the V3-V4 hypervariable region of the 16S rRNA gene was carried out using universal primers (341F: 5′-CCTACGGGNGGCWGCAG-3′ and 805R: 5′-GACTACHVGGGTATCTAATCC-3′). The PCR reaction was conducted in a 30 μL reaction system, which included 15 μL of 2× KAPA HiFi Mix (KAPA Biosystems, Wilmington, MA, United States), 1 μL each of the forward and reverse primers, and 12.5 ng of template DNA. The PCR conditions were as follows: 95°C for 3 min, followed by 25 cycles of 95°C for 30 s, 55°C for 30 s, and 72°C for 15 s, with a final extension at 72°C for 5 min, and storage at 4°C. The concentration of the PCR products, purified with AMPure XT magnetic beads (Beckman Coulter Genomics, Danvers, MA, USA), was measured using the Invitrogen Qubit 4 System (Thermo Fisher Scientific Inc., Wilmington, NC, USA). The final products were then checked using 1.5% agarose gel electrophoresis. The mixed PCR products were then used for library construction, with sequencing adapters added and library index information recorded. Reaction conditions: Pre-denaturation at 98°C for 45 s; Denaturation at 98°C for 15 s; Annealing at 60°C for 30 s; Extension at 72°C for 30 s, repeated for eight cycles; Final extension at 72°C for 10 min, followed by storage at 4°C. After PCR amplification, primer dimers and other small fragments were removed using AMPure XT magnetic beads. Prior to sequencing, the library’s concentration was quantified and calculated using Qubit. The validated library was sequenced on the Illumina MiSeq platform (Illumina, San Diego, CA, USA), generating 2 × 250 bp paired-end reads.

### 2.4 Statistical analysis and bioinformatics

The raw sequence primers were trimmed using QIIME2 and then quality filtered using DADA2 (version 1.29.0) with the default parameters. The output file was the amplicon sequence variant (ASV). The ASVs were then mapped to the Silva reference database (version 138) for taxonomic annotation. Alpha diversity indices, including community richness, community diversity, and community evenness, were assessed using QIIME2. Beta diversity, which demonstrates differences in the compositional structure of microbial communities between subgroups, was assessed using Principal Coordinate Analysis (PCoA) with the Bray-Curtis, Jaccard, Unweighted-Unifrac, and Weighted-Unifrac algorithms. The PICRUSt2 software, using default parameters, generated predicted genomes based on ASV files and compared them to the KEGG database to identify potential gene functions in the GM. The comparison of potential gene functions in each group was analyzed using the Kruskal–Wallis test. Linear discriminant analysis effect sizes (LEfSe) were used to identify unique bacterial taxa (log LDA score >2 and *p* < 0.05) for comparison. R language (version 4.2.1) and ggplot2 were used to visualize the analysis. In addition, the construction of the Interactive Venn diagram was done using the method of Chen et al. (2021). Data analysis was performed using SPSS Statistics (version 23.0), and all values are expressed as mean ± standard deviation (SD). A *p*-value of less than 0.05 was considered to indicate a significant difference.

## 3 Results

### 3.1 Demographic data

A total of 25 serious runners and 22 healthy individuals participated in this study, with demographic data shown in Table 1. The *t*-test results indicated no significant differences in age and BMI between the RG and CG groups, but there was a significant difference in heart rate and weekly exercise volume (*p* < 0.0001). Furthermore, the likelihood ratio chi-square test based on dietary composition data showed no significant differences in dietary patterns between the two groups.

**TABLE 1:** Participant characteristics.

	RG (N = 25)	CG (N = 22)	*P* ^a^
Gender (Male/Female)	13/12	8/14	
Age	49 ± 3.75	49.23 ± 3.13	0.824
BMI	22.14 ± 1.98	22.66 ± 1.89	0.371
Heart rate (beats/min)	57.4 ± 4.65	73 ± 4.41	<0.0001
Km/week	46.45 ± 3.24	3.7 ± 2.35	<0.0001
Dietary Habits (n)			*P* ^b^
Dairy products (Fr/S/O/N)	7/6/8/4	5/8/5/4	0.770
Fried and fatty food (Fr/S/O/N)	2/3/7/13	2/3/8/9	0.895
Refined carbohydrates (Fr/S/O/N)	3/2/17/3	1/3/16/2	0.732
High protein foods (Fr/S/O/N)	24/1/0/0	17/3/2/0	0.089
Fruits and vegetables (Fr/S/O/N)	22/3/0/0	18/3/1/0	0.450
Protein supplementation (Fr/S/O/N)	1/5/7/12	0/5/6/11	0.726

^a^ *t*-test.

^b^ likelihood ratio chi-square test.

### 3.2 Gut microbiota (GM) diversity

**FIGURE 1:**
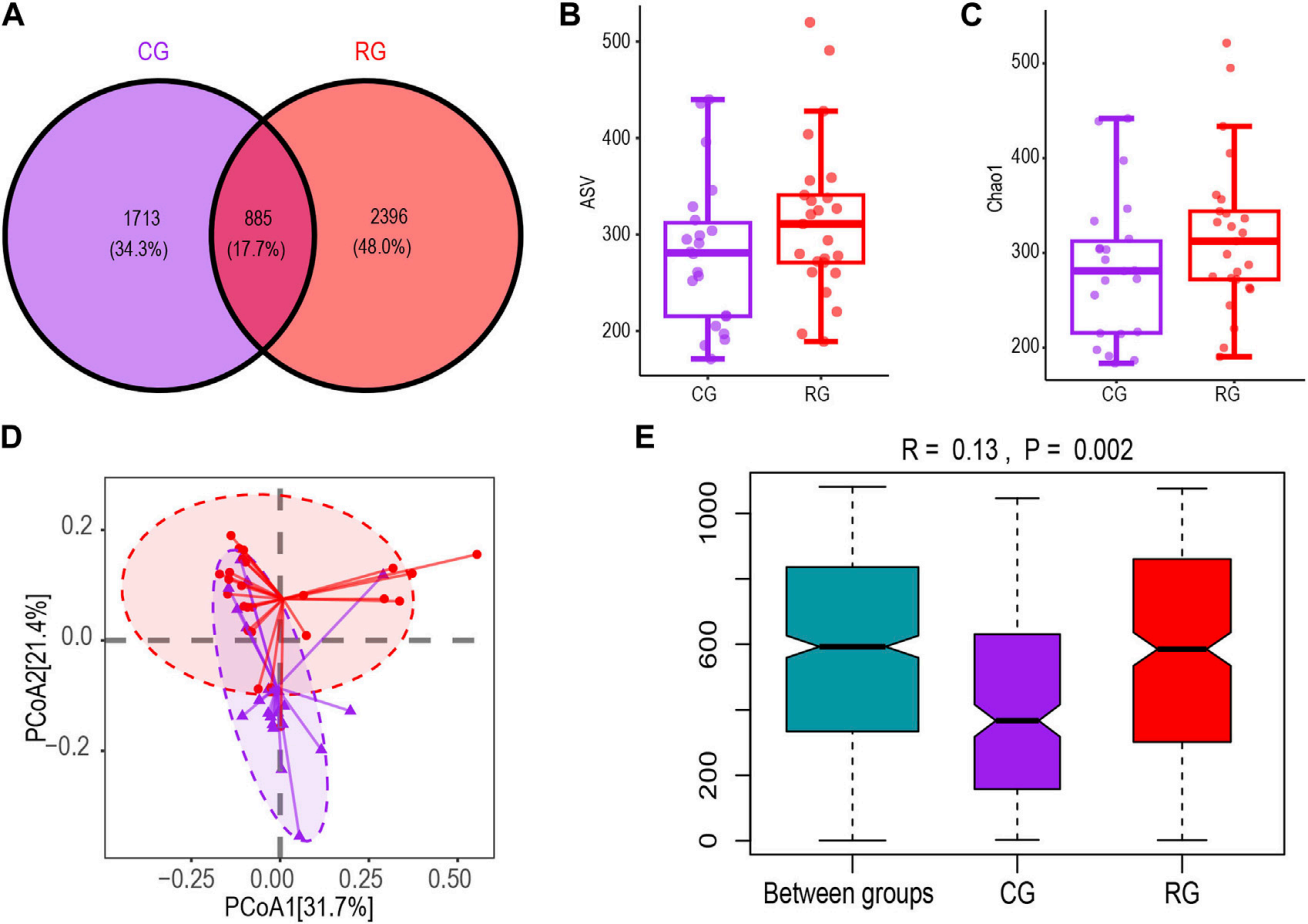
Diversity characteristics of the intestinal microbiota of serious runners group (RG) (n = 25) and healthy control group (CG) (n = 22). **(A,B)** represent the Venn diagram and box scatter plot based on the number of ASVs respectively; **(C)** Chao one index. **(D)** PCoa results based on the unweighted UniFrac distance algorithm. The horizontal and vertical coordinates respectively represent the two dimensions explaining the maximum proportion of variance in the community. **(E)** Between- and within-group differences in CG and RG based on one-way ANOSIM assessment. The boxplots from left to right represent the unweighted UniFrac distance between RG and CG samples, RG samples, and CG samples respectively. R values and *p* values show community changes between compared groups.

We obtained 7,920,375 reads of the 16S rRNA gene from a total of 47 fecal samples. After quality filtering, 4,282,406 high-quality reads were obtained. Each sample’s average read count was 91,115. A total of 4,994 ASVs were obtained for analysis, with each sample observing 173–520 ASVs (Supplementary Table S1). The Venn diagram of ASV differences showed that RG and CG shared 885 ASVs. RG had more unique ASVs than CG, but this difference was not statistically significant (Figures 1A, B). The boxplot based on the Chao1 index showed no significant difference in α diversity between CG and RG (Figure 1C). PCoA clustering analysis and ANOSIM based on unweighted UniFrac distance were used to characterize the differences in microbial community spatial structure between groups. The PCoA clustering results showed that RG and CG clustered separately (Figure 1D). The ANOSIM results (R = 0.13, *p* = 0.002) indicated that the inter-group differences were significant and greater than the intra-group differences (Figure 1E).

### 3.3 Differences in the composition of gut microbiota

**FIGURE 2:**
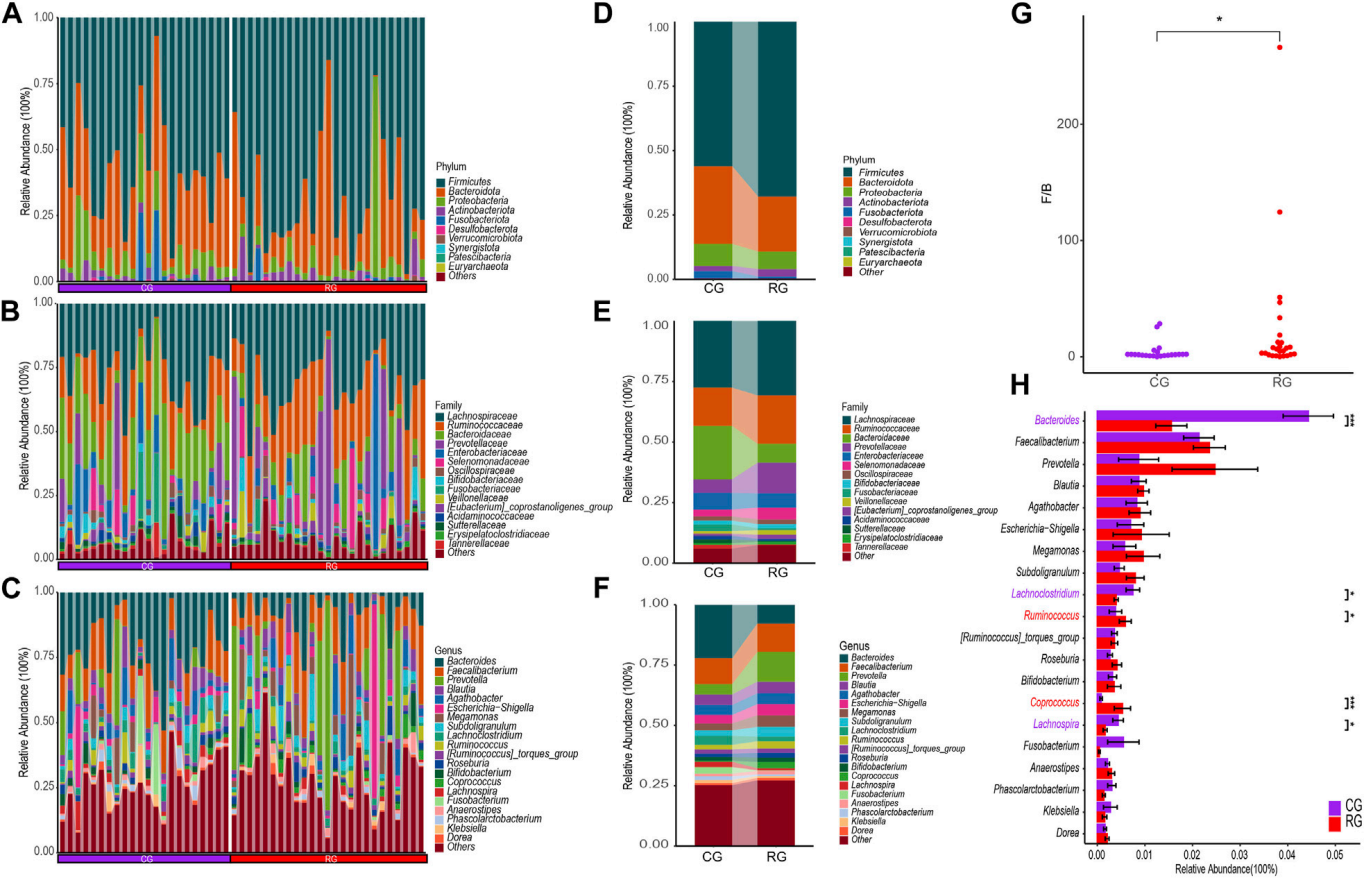
Differential analysis of gut microbiota composition in the serious runners group (RG) and healthy control group (CG). **(A–C)** represent the relative abundance of all samples at the phylum (top 10), family (top 15), and genus (top 20) levels, respectively. Each color represents a taxonomic group and its name is shown on the right side of figure. **(D–F)** represent the average relative abundance at the level of phylum (top 10), family (top 15), and genus (top 20) in RG and CG, respectively. **(G)** Box scatter plots of RG and CG groups based on the ratio of *Firmicutes/Bacteroidota*. **(H)** Difference bars for the top 20 genera in terms of relative abundance between the RG and CG groups. *, *p* < 0.05; **, *p* < 0.01; ***, *p* < 0.001.

Through the SILVA database, a total of 12 phyla, 17 classes, 44 orders, 76 families, and 233 genera were obtained (Figures 2A–C). At the phylum level, the top three phyla in relative abundance in RG and CG were *Firmicutes, Bacteroidota,* and *Proteobacteria* (Figure 2D). *Firmicutes* were relatively enriched in RG while *Bacteroidota* were relatively decreased, resulting in a significant difference in the *Firmicutes/Bacteroidota* ratio between CG and RG (Figure 2G). At the family level, *Lachnospiraceae, Ruminococcaceae, Prevotellaceae, Bacteroidaceae,* and *Enterobacteriaceae* were significantly enriched in both groups. Compared to CG, the changes in RG were primarily characterized by a notable decrease in *Bacteroidaceae* and an increase in the abundance of *Lachnospiraceae, Ruminococcaceae,* and *Prevotellaceae* (Figure 2E). At the genus level, the primary advantages of the two groups are *Prevotella, Faecalibacterium, Bacteroides*, and *Blautia* (Figure 2F). Among the top 20 genera in terms of average relative abundance, *Ruminococcus* and *Coprococcus* are significantly enriched in RG, while *Bacteroides, Lachnoclostridium*, and *Lachnospira* are significantly enriched in CG (Figure 2H).

**FIGURE 3:**
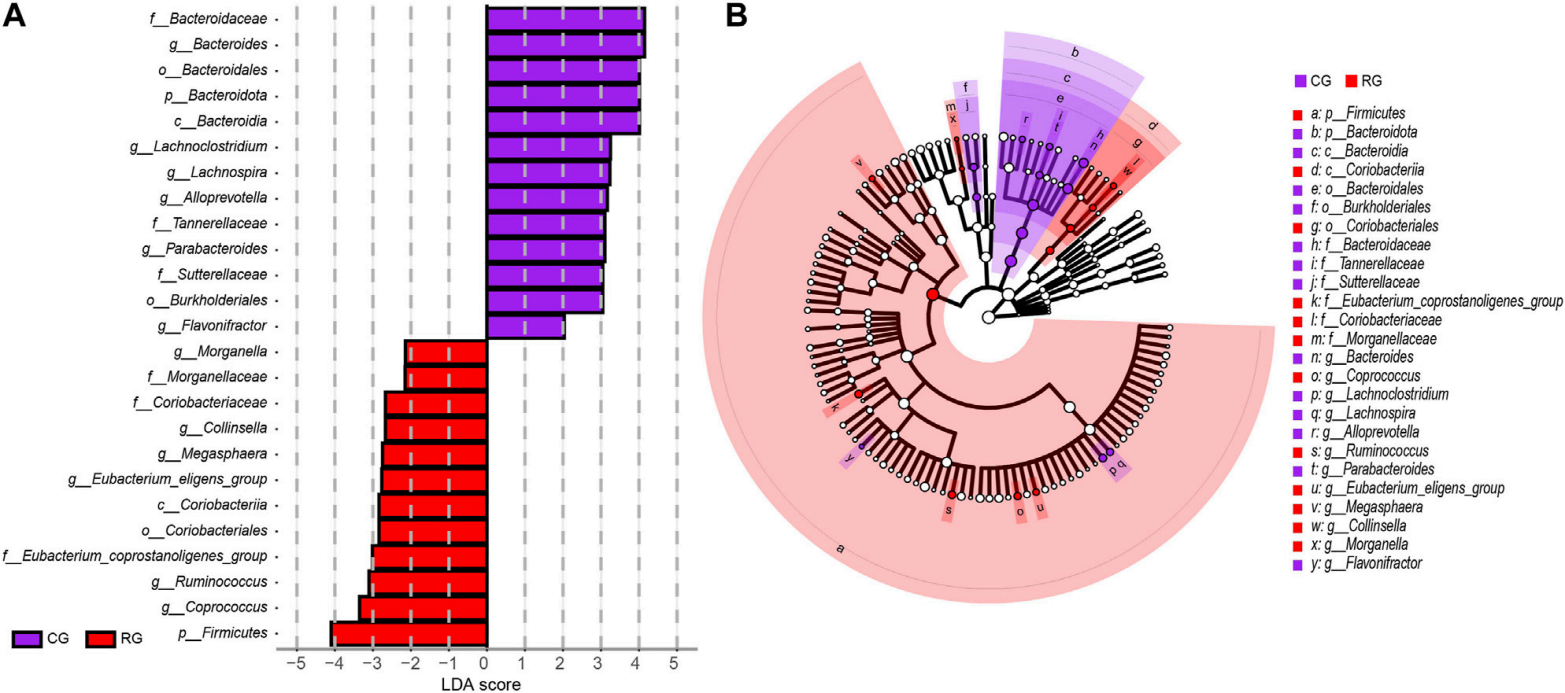
Linear discriminant analysis effect size (LEfSe) analysis of the different structures of the gut microbiota in the serious runners group (RG) and healthy controls group (CG). **(A)** Taxonomic groups showing LDA scores >2.0 with *p* < 0.05. **(B)** Cladogram of significant changes at all taxonomic levels. The root of the cladogram represents the domain bacteria, and the size of the node represents the abundance of the taxa in the cladogram.

The analysis results of LEfSe further identified specific differential bacterial biomarkers in the GM of CG and RG (Figure 3; Supplementary Table S2). Linear discriminant analysis (LDA) results revealed 25 bacteria with differential relative abundance between the two groups, with 12 enriched in RG, mainly included *Firmicutes, Eubacterium_coprostanoligenes_group, Coprococcus,* and *Ruminococcus*. Additionally, the 13 differentially enriched bacteria in CG mainly included *Bacteroidota, Bacteroidia, Bacteroidales, Bacteroidaceae*, and *Bacteroides.*

### 3.4 Correlation between gut microbiota and predicted functional pathways

**FIGURE 4:**
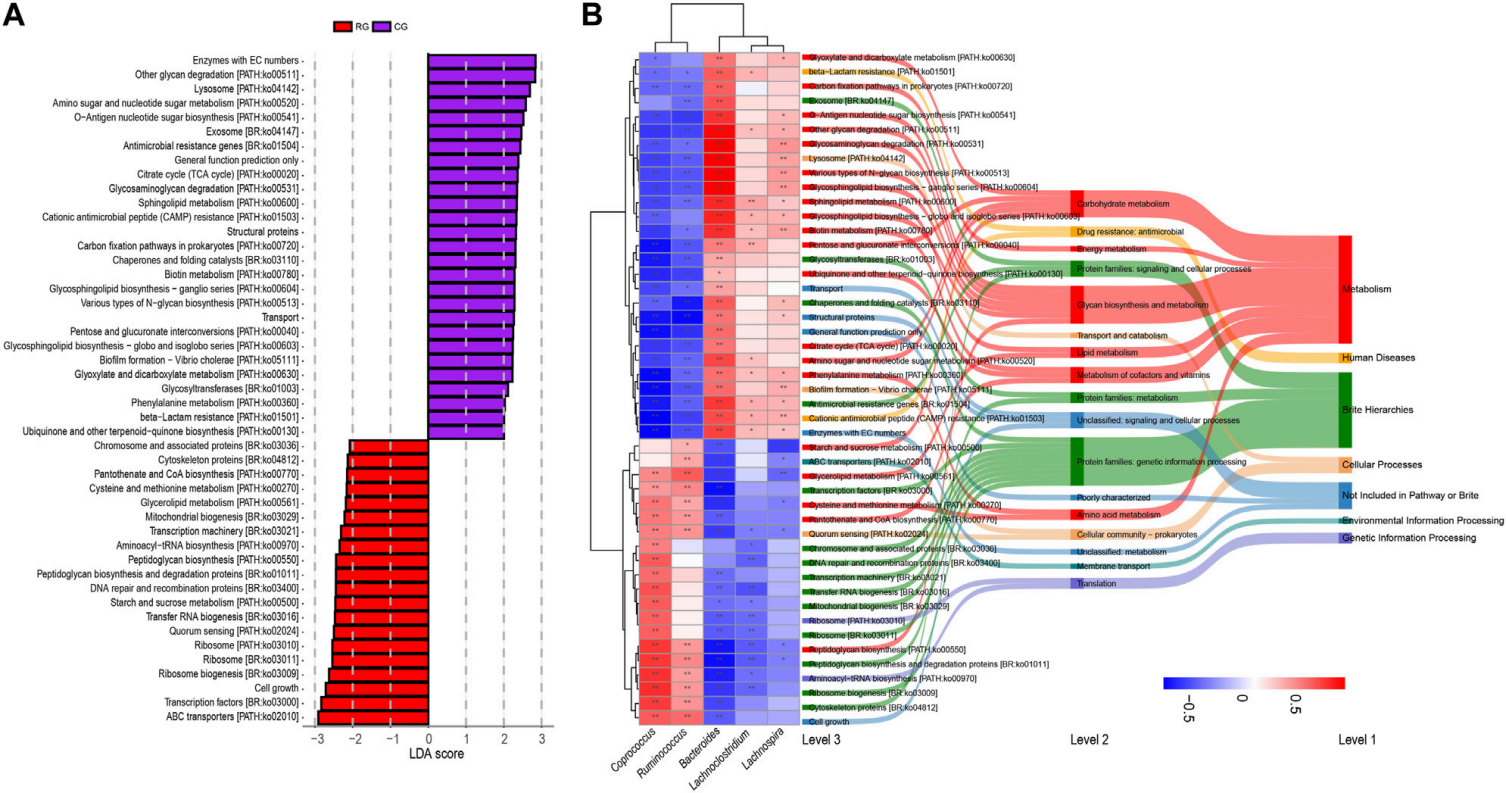
**(A)** Linear discriminant analysis effect size (LEfSe) analysis of KEGG level 3 functional pathway in the gut microbiota of serious runners (RG) and healthy controls (CG) with different structures. Categorical group shows LDA score >2.0, *p* < 0.05. **(B)** Spearman correlation analysis based on five differential genera and 47 differential KEGG level3 pathways. From left to right, correlation clustering heatmaps, KEGG level3 pathways, KEGG level2 pathways, and KEGG level1 pathways. The heat map color blocks are coded according to the correlation coefficients, from red to blue in descending order. The KEGG three-level pathways are uniformly colored according to level 1. The feature clustering pattern is presented by a dendrogram on the left side of the heat map. *, *p* < 0.05; **, *p* < 0.01.

Functional prediction of GM composition was performed for different samples using PICRUSt2 software. By comparing with the KEGG database, we identified a total of 51 secondary pathways. The top three pathways in terms of average relative abundance were carbohydrate metabolism (9.83%), energy metabolism (3.92%), and lipid metabolism (1.84%). Among the 407 tertiary pathways, 47 pathways showed significant differences in relative abundance between RG and CG (*p* < 0.05 in LEfSe analysis, LDA score >2, Figure 4; Supplementary Table S3), including 20 in metabolism, and others in cellular processes (3), human diseases (2), genetic information processing (2), and environmental information processing (1). Next, we conducted Spearman analysis on the five major differentially abundant genera obtained earlier against these 47 differential functional pathways. The results indicate that the bacteria enriched in RG and CG exhibit both intra-group similarity and inter-group differences in terms of functionality. The differentially enriched genera in RG are positively correlated with differential metabolic pathways related to cellular processes, environmental information processing, and genetic information processing, while most differential pathways related to metabolism and human diseases show a negative correlation. Only a few metabolism-related pathways (starch and sucrose metabolism, glycerolipid metabolism, cysteine and methionine metabolism, pantothenate and CoA biosynthesis, peptidoglycan biosynthesis) are enriched in RG.

## 4 Discussion

Exercise can be regarded as an immune system adjuvant, a behavior that promotes a healthy host and contributes to metabolism while improving immunity (Huang et al., 2013; Nieman and Wentz, 2019). In contrast, lack of physical activity increases the risk of developing a variety of chronic diseases, including metabolic disorders, cardiovascular diseases, neurological disorders, and other inflammatory-related diseases (Gleeson et al., 2011). In addition, as life expectancy increases, the body’s immune and metabolic systems may become dysfunctional, leading to the development of various diseases. Moderate exercise helps reduce or delay this dysfunction and achieve a state of immune remodeling (Warburton and Bredin, 2017). The “exercise factor” is currently recognized as a key factor in exercise-related health, acting mainly through endocrine, paracrine, and/or autocrine pathways (Gleeson et al., 2011; Nieman, 2012; Chow et al., 2022). GM also plays an important role in this process. In addition to maintaining the gut barrier, some of these microorganisms can benefit the host by metabolizing beneficial components, such as glycans, amino acids, vitamins, and short-chain fatty acids (Francino, 2016; Santacroce et al., 2021). Exercise can modify the composition and structure of the gut microbes through various mechanisms. These mechanisms include increasing the body temperature of the host and upregulating anti-inflammatory cytokines and antioxidant enzymes (Mailing et al., 2019). Interestingly, gut microbes can also influence host motility through the gut-brain axis pathway (Dohnalová et al., 2022). Such metabolic differences in the exercising population compared to healthy controls depend on structural and compositional alterations in the GM.

In our study, although there was no significant difference in the alpha diversity of GM between the RG and CG groups, the beta diversity results suggested that the composition of GM in the RG was distinct from that in the CG. Previous studies have shown that exercise is beneficial for the diversity of GM, and this effect appears to be more commonly reported in professional athletes (Clarke et al., 2014; Petersen et al., 2017; Barton et al., 2018; Keohane et al., 2019; Liang et al., 2019). Research in the elderly population has shown that the impact of exercise on GM primarily leads to significant differences in beta diversity rather than alpha diversity (Fart et al., 2020; Šoltys et al., 2021). Therefore, characterizing the impact of exercise on GM diversity based solely on diversity may be limited and could be influenced by factors such as sample size. Although there is some controversy over the impact of exercise on the diversity of GM, it is undeniable that the GM composition in all exercise-related groups differs from that of the control group. *Firmicutes* and *Bacteroidota* are the main phyla of GM, and changes in the *Firmicutes/Bacteroidota* ratio (F/B) are thought to be associated with obesity (Ley et al., 2005). However, this viewpoint seems to be controversial at present (Walters et al., 2014; Walker and Hoyles, 2023). Similarly, there are conflicting reports on the correlation between the F/B ratio and physical fitness indicators (e.g., VO_2_max) in exercise studies (Hughes, 2020). Research by Kulecka et al. (2020) showed an increased F/B ratio in marathon runners and cross-country skiers, while Druk et al. (Durk et al., 2019) found a significant positive correlation between the F/B ratio and VO_2_max. On the contrary, the research results of Yu et al. (Yu et al., 2018) show that older adults with higher physical abilities have lower F/B ratios. Nevertheless, these results all suggest a potential association between the F/B ratio and physical fitness indicators. In our study, although we also observed a higher F/B ratio in RG, this difference was primarily influenced by a small number of individuals, indicating a relatively weak significant difference in our study population. According to the theory proposed by Estaki et al. (2016), the correlation between exercise-related cardiopulmonary health and the gut microbiota is more evident in functionality rather than taxonomic groups. This also reasonably explains seemingly contradictory results in some studies. However, the specific mechanism still requires further investigation. The significantly enriched genera *Ruminococcus* and *Coprococcus* in the RG may benefit host health. *Ruminococcus* was identified as a distinguishing characteristic of elite athletes in a study conducted by Han et al. (2020). This is believed to be linked to the increased muscle mass (Donati Zeppa et al., 2021). *Ruminococcus* is a genus found in the thick-walled homopteran family that has the ability to produce butyric acid, which can boost immunity and maintain host health (Wang et al., 2017). Similarly, *Coprococcus* has the potential to produce butyric acid. In a recent report, *Coprococcus eutactus* was identified as a potential probiotic for improving colitis (Yang et al., 2023). Furthermore, significant differences were observed between the athletic and non-athletic populations of *Coprococcus*, with intense endurance exercise leading to a rapid increase in the abundance of this genus (Bressa et al., 2017; Zhao et al., 2018; Moitinho-Silva et al., 2021). It is important to note that the presence of this beneficial host microorganism can be achieved not only through regular exercise but also through dietary modifications and nutritional interventions such as probiotic and vitamin supplementation. These interventions can also enhance the abundance of *Coprococcus* spp. and improve the overall health of the host (Notting et al., 2023).

The composition and metabolism of the GM are closely related (Barton et al., 2015). Functional predictions based on PICRUST indicate that serious runners have more genetic information processing functions (such as transcription factors, peptidoglycan biosynthesis and degradation proteins, and ribosome biogenesis), as well as functions related to membrane transport and translation. These functional pathways are positively correlated with the enrichment of *Ruminococcus* and *Coprococcus* in the gut. This suggests that exercise may promote the synthesis and turnover of bacterial DNA and proteins by altering the composition of the GM, similar to the results reported by Taniguchi et al.(Taniguchi et al., 2018) During exercise, gut bacteria play a role in maintaining gut microbial balance (Wegierska et al., 2022). They can enhance exercise performance by promoting carbohydrate metabolism and energy metabolism related to the energy required for exercise (Jeukendrup, 2014; Liang et al., 2019). In our study, most of the differential pathways related to carbohydrate metabolism and glycan biosynthesis and metabolism are enriched in CG. In the carbohydrate metabolism pathway, only the starch and sucrose metabolism pathway is significantly increased in RG. This pathway is closely related to glucose metabolism and energy supply, indicating that starch and sucrose metabolism may be more susceptible to the influence of exercise. In addition, *Bacteroides,* as the most common bacterial genus in the human gut, shows strong correlations with the majority of differential functional pathways, especially those related to glycan biosynthesis and metabolism. This association is closely linked to its ability to produce surface glycans (Coyne et al., 2008). It is worth noting that the peptidoglycan biosynthesis enriched in RG shows a significant negative correlation with *Bacteroides.* Peptidoglycan, an immunogenic substance, is abundant in the GM and plays a role in regulating immune and inflammatory responses. Laman et al. suggest that bacterial peptidoglycan could be a significant contributing factor to chronic brain inflammation (Laman et al., 2020). However, Yin et al.'s recent review, emphasizing the significance of the gut microbiome in reshaping immune responses, underscores the potential advantages of immunogenic molecules derived from gut bacteria in treating infections and immune diseases (Yin et al., 2023). Although the potential mechanisms linking exercise, GM, and host metabolism are not yet clear, it is undeniable that a healthy GM environment contributes to maintaining the host’s healthy physiological metabolism.

The study was limited by the relatively small number of participants, while dietary patterns were undeniably a significant influence. Most of the volunteers selected from the long distance running association exhibited relatively good self-regulated eating behaviors, particularly in the middle-aged group. However, most of the middle-aged group had chronic diseases, and there were few healthy subjects who met the criteria for NAR, especially in the control group.

Compared to the healthy control group, serious runners exhibit significant differences in the composition of their GM as well as in the predicted outcomes of their functional pathways. A higher F/B ratio and a high abundance of *Ruminococcus* and *Coprococcus* are significant characteristics of serious runners. Although no significant differences were found in dietary patterns statistically, it is important to note that this assessment may be influenced by recall bias. Therefore, in addition to expanding the sample size, strict control of dietary patterns should be included in future studies. Furthermore, the integrated analysis of metagenomics and metabolomics along with additional physiological data will help to better understand the potential connections between exercise, GM, and health.

## Data Availability

The datasets presented in this study can be found in online repositories. The names of the repository/repositories and accession number(s) can be found in the article/Supplementary Material.
