# Supplementary material for: The impact of exercise on the gut microbiota in middle-aged amateur serious runners: a comparative study
Source: Front Physiol. 2024 Apr 26;15:1343219. doi: 10.3389/fphys.2024.1343219 (PMC11082653; doi:10.3389/fphys.2024.1343219)
Supplement: Supplementary file 1 [file Table1.docx]

**Table S1.** 16S rRNA data from fecal samples.

| **SampleID** | **Accession number** | **Raw reads** | **Hlgh quality reads** | **ASV** | **R** | **Sum length** | **Minimum sequence length** | **Average sequence length** | **Maximum sequence length** |
| --- | --- | --- | --- | --- | --- | --- | --- | --- | --- |
| S0187693329 | SAMN38256198 | 168974 | 100885 | 259 | R1 | 38353621 | 64 | 227 | 234 |
|  |  |  |  |  | R2 | 38007519 | 67 | 224.9 | 229 |
| S0187695210 | SAMN38256199 | 182534 | 94964 | 256 | R1 | 41433637 | 112 | 227 | 232 |
|  |  |  |  |  | R2 | 41065857 | 112 | 225 | 230 |
| S0187696985 | SAMN38256200 | 161411 | 117367 | 206 | R1 | 36639323 | 0 | 227 | 232 |
|  |  |  |  |  | R2 | 36317248 | 0 | 225 | 235 |
| S0187697081 | SAMN38256165 | 154286 | 78857 | 361 | R1 | 35021766 | 5 | 227 | 232 |
|  |  |  |  |  | R2 | 34713607 | 5 | 225 | 234 |
| S0187697135 | SAMN38256178 | 183513 | 137143 | 239 | R1 | 41650205 | 0 | 227 | 232 |
|  |  |  |  |  | R2 | 41286637 | 0 | 225 | 234 |
| S0187697909 | SAMN38256179 | 162218 | 68926 | 275 | R1 | 36821556 | 45 | 227 | 233 |
|  |  |  |  |  | R2 | 36498654 | 45 | 225 | 229 |
| S0187697930 | SAMN38256201 | 148853 | 75099 | 281 | R1 | 33784997 | 64 | 227 | 232 |
|  |  |  |  |  | R2 | 33476658 | 26 | 224.9 | 231 |
| S0187698179 | SAMN38256166 | 146312 | 75227 | 340 | R1 | 33207957 | 49 | 227 | 233 |
|  |  |  |  |  | R2 | 32916201 | 41 | 225 | 232 |
| S0187698614 | SAMN38256180 | 175852 | 78047 | 491 | R1 | 39901064 | 67 | 226.9 | 230 |
|  |  |  |  |  | R2 | 39551698 | 118 | 224.9 | 229 |
| S0187698665 | SAMN38256167 | 182416 | 77183 | 335 | R1 | 41406082 | 122 | 227 | 233 |
|  |  |  |  |  | R2 | 41041718 | 26 | 225 | 231 |
| S0187698716 | SAMN38256202 | 181368 | 102748 | 330 | R1 | 41168453 | 64 | 227 | 233 |
|  |  |  |  |  | R2 | 40801997 | 37 | 225 | 232 |
| S0187698766 | SAMN38256203 | 169087 | 92116 | 290 | R1 | 38379383 | 116 | 227 | 234 |
|  |  |  |  |  | R2 | 38040939 | 37 | 225 | 229 |
| S0187698873 | SAMN38256204 | 175965 | 82988 | 214 | R1 | 39942772 | 97 | 227 | 234 |
|  |  |  |  |  | R2 | 39590125 | 34 | 225 | 230 |
| S0187698985 | SAMN38256190 | 181529 | 103354 | 303 | R1 | 41202664 | 49 | 227 | 234 |
|  |  |  |  |  | R2 | 40839538 | 49 | 225 | 230 |
| S0187699051 | SAMN38256205 | 152637 | 76532 | 281 | R1 | 34646039 | 15 | 227 | 232 |
|  |  |  |  |  | R2 | 34339651 | 15 | 225 | 233 |
| S0187699072 | SAMN38256181 | 147666 | 73410 | 321 | R1 | 33517830 | 122 | 227 | 232 |
|  |  |  |  |  | R2 | 33218329 | 34 | 225 | 231 |
| S0187699161 | SAMN38256206 | 163601 | 100940 | 345 | R1 | 37125133 | 34 | 226.9 | 233 |
|  |  |  |  |  | R2 | 36798825 | 34 | 224.9 | 230 |
| S0187699273 | SAMN38256182 | 162259 | 94910 | 279 | R1 | 36830490 | 1 | 227 | 234 |
|  |  |  |  |  | R2 | 36504287 | 1 | 225 | 230 |
| S0187701797 | SAMN38256183 | 163630 | 78729 | 328 | R1 | 37142846 | 120 | 227 | 234 |
|  |  |  |  |  | R2 | 36814927 | 26 | 225 | 231 |
| S0187702143 | SAMN38256184 | 161294 | 84685 | 261 | R1 | 36611627 | 130 | 227 | 232 |
|  |  |  |  |  | R2 | 36290490 | 34 | 225 | 229 |
| S0187702624 | SAMN38256191 | 170476 | 101669 | 216 | R1 | 38697138 | 104 | 227 | 233 |
|  |  |  |  |  | R2 | 38349431 | 110 | 225 | 231 |
| S0187702663 | SAMN38256192 | 174779 | 91403 | 299 | R1 | 39673678 | 67 | 227 | 233 |
|  |  |  |  |  | R2 | 39324066 | 34 | 225 | 231 |
| S0187702791 | SAMN38256168 | 177956 | 89562 | 357 | R1 | 40384669 | 67 | 226.9 | 234 |
|  |  |  |  |  | R2 | 40026867 | 100 | 224.9 | 229 |
| S0187702985 | SAMN38256185 | 151414 | 77716 | 273 | R1 | 34369891 | 130 | 227 | 232 |
|  |  |  |  |  | R2 | 34067987 | 122 | 225 | 233 |
| S0187703268 | SAMN38256169 | 181488 | 125352 | 198 | R1 | 41197261 | 49 | 227 | 234 |
|  |  |  |  |  | R2 | 40834907 | 49 | 225 | 228 |
| S0187703283 | SAMN38256193 | 170182 | 111172 | 187 | R1 | 38628223 | 11 | 227 | 234 |
|  |  |  |  |  | R2 | 38289160 | 11 | 225 | 230 |
| S0187703401 | SAMN38256207 | 164792 | 95724 | 173 | R1 | 37406552 | 64 | 227 | 234 |
|  |  |  |  |  | R2 | 37077880 | 64 | 225 | 229 |
| S0187703418 | SAMN38256170 | 146469 | 75424 | 271 | R1 | 33246105 | 15 | 227 | 233 |
|  |  |  |  |  | R2 | 32952693 | 15 | 225 | 233 |
| S0187703572 | SAMN38256194 | 169352 | 85969 | 394 | R1 | 38441053 | 94 | 227 | 234 |
|  |  |  |  |  | R2 | 38102669 | 1 | 225 | 229 |
| S0187703579 | SAMN38256171 | 175782 | 81079 | 294 | R1 | 39900680 | 122 | 227 | 234 |
|  |  |  |  |  | R2 | 39548874 | 34 | 225 | 229 |
| S0187703603 | SAMN38256186 | 165123 | 79398 | 261 | R1 | 37481377 | 9 | 227 | 233 |
|  |  |  |  |  | R2 | 37150964 | 9 | 225 | 231 |
| S0187703616 | SAMN38256172 | 217113 | 98814 | 520 | R1 | 49282217 | 41 | 227 | 235 |
|  |  |  |  |  | R2 | 48847271 | 34 | 225 | 230 |
| S0187704146 | SAMN38256195 | 189786 | 88297 | 197 | R1 | 43075469 | 47 | 227 | 234 |
|  |  |  |  |  | R2 | 42697300 | 47 | 225 | 229 |
| S0187704561 | SAMN38256187 | 165035 | 74258 | 311 | R1 | 37460097 | 45 | 227 | 233 |
|  |  |  |  |  | R2 | 37130972 | 15 | 225 | 231 |
| S0187704675 | SAMN38256208 | 170648 | 111154 | 296 | R1 | 38722489 | 5 | 226.9 | 233 |
|  |  |  |  |  | R2 | 38382627 | 5 | 224.9 | 229 |
| S0187704687 | SAMN38256209 | 190388 | 110316 | 263 | R1 | 43212391 | 47 | 227 | 234 |
|  |  |  |  |  | R2 | 42826999 | 49 | 224.9 | 233 |
| S0187704724 | SAMN38256173 | 169242 | 107776 | 221 | R1 | 38415661 | 15 | 227 | 232 |
|  |  |  |  |  | R2 | 38077710 | 15 | 225 | 230 |
| S0187707549 | SAMN38256196 | 173157 | 78765 | 191 | R1 | 39304726 | 30 | 227 | 233 |
|  |  |  |  |  | R2 | 38959020 | 30 | 225 | 228 |
| S0187710475 | SAMN38256174 | 162413 | 90103 | 338 | R1 | 36866857 | 30 | 227 | 232 |
|  |  |  |  |  | R2 | 36540821 | 30 | 225 | 230 |
| S0187710632 | SAMN38256175 | 171311 | 86459 | 186 | R1 | 38877734 | 120 | 226.9 | 234 |
|  |  |  |  |  | R2 | 38535535 | 122 | 224.9 | 228 |
| S0187710998 | SAMN38256188 | 150656 | 74202 | 280 | R1 | 34197157 | 49 | 227 | 233 |
|  |  |  |  |  | R2 | 33895270 | 49 | 225 | 228 |
| S0187711029 | SAMN38256210 | 153265 | 79652 | 440 | R1 | 34787512 | 15 | 227 | 234 |
|  |  |  |  |  | R2 | 34477033 | 15 | 225 | 229 |
| S0187711072 | SAMN38256197 | 163318 | 81371 | 315 | R1 | 37069075 | 122 | 227 | 233 |
|  |  |  |  |  | R2 | 36742647 | 34 | 225 | 232 |
| S0187711113 | SAMN38256189 | 170854 | 138939 | 321 | R1 | 38781268 | 17 | 227 | 234 |
|  |  |  |  |  | R2 | 38440227 | 15 | 225 | 232 |
| S0187715676 | SAMN38256176 | 186331 | 93802 | 428 | R1 | 42294820 | 122 | 227 | 232 |
|  |  |  |  |  | R2 | 41898686 | 122 | 224.9 | 228 |
| S0187715749 | SAMN38256211 | 170145 | 86215 | 433 | R1 | 38611181 | 1 | 226.9 | 234 |
|  |  |  |  |  | R2 | 38273165 | 1 | 224.9 | 231 |
| S0187716009 | SAMN38256177 | 143495 | 73705 | 405 | R1 | 32572608 | 15 | 227 | 232 |
|  |  |  |  |  | R2 | 32285749 | 15 | 225 | 232 |

**Table S2.** Lefse analysis results based on species composition.

| **Biomarker names** | **Log value** | **Group** | **LDA score** | ***p* value** |
| --- | --- | --- | --- | --- |
| *k__Bacteria\|p__Firmicutes* | 5.132588079 | RG | 4.09938125 | 0.03129297 |
| *k__Bacteria\|p__Firmicutes\|c__Clostridia\|o__Lachnospirales\|f__Lachnospiraceae\|g__Coprococcus* | 3.722522863 | RG | 3.351876836 | 0.00028915 |
| *k__Bacteria\|p__Firmicutes\|c__Clostridia\|o__Oscillospirales\|f__Eubacterium_coprostanoligenes_group* | 3.548229711 | RG | 3.013312274 | 0.042825609 |
| *k__Bacteria\|p__Firmicutes\|c__Clostridia\|o__Oscillospirales\|f__Ruminococcaceae\|g__Ruminococcus* | 3.768742754 | RG | 3.103525351 | 0.029656556 |
| *k__Bacteria\|p__Actinobacteriota\|c__Coriobacteriia* | 3.329203761 | RG | 2.839771782 | 0.004574406 |
| *k__Bacteria\|p__Actinobacteriota\|c__Coriobacteriia\|o__Coriobacteriales* | 3.329203761 | RG | 2.839771782 | 0.004574406 |
| *k__Bacteria\|p__Firmicutes\|c__Clostridia\|o__Lachnospirales\|f__Lachnospiraceae\|g__Eubacterium_eligens_group* | 3.307633625 | RG | 2.7670174 | 0.008414536 |
| *k__Bacteria\|p__Actinobacteriota\|c__Coriobacteriia\|o__Coriobacteriales\|f__Coriobacteriaceae* | 3.124970271 | RG | 2.671381503 | 0.004785804 |
| *k__Bacteria\|p__Firmicutes\|c__Negativicutes\|o__Veillonellales_Selenomonadales\|f__Veillonellaceae\|g__Megasphaera* | 3.106179098 | RG | 2.74668122 | 0.000821063 |
| *k__Bacteria\|p__Proteobacteria\|c__Gammaproteobacteria\|o__Enterobacterales\|f__Morganellaceae* | 2.36224554 | RG | 2.153148679 | 0.026962508 |
| *k__Bacteria\|p__Actinobacteriota\|c__Coriobacteriia\|o__Coriobacteriales\|f__Coriobacteriaceae\|g__Collinsella* | 3.124970271 | RG | 2.671381503 | 0.004785804 |
| *k__Bacteria\|p__Proteobacteria\|c__Gammaproteobacteria\|o__Enterobacterales\|f__Morganellaceae\|g__Morganella* | 2.36224554 | RG | 2.145487737 | 0.007341561 |
| *k__Bacteria\|p__Bacteroidota\|c__Bacteroidia\|o__Bacteroidales\|f__Bacteroidaceae* | 4.646562689 | CG | 4.146474022 | 6.70E-05 |
| *k__Bacteria\|p__Bacteroidota\|c__Bacteroidia\|o__Bacteroidales\|f__Bacteroidaceae\|g__Bacteroides* | 4.646562689 | CG | 4.146474022 | 6.70E-05 |
| *k__Bacteria\|p__Bacteroidota\|c__Bacteroidia\|o__Bacteroidales* | 4.780436649 | CG | 4.014118486 | 0.026603455 |
| *k__Bacteria\|p__Bacteroidota* | 4.780460414 | CG | 4.01409959 | 0.026603455 |
| *k__Bacteria\|p__Bacteroidota\|c__Bacteroidia* | 4.780460414 | CG | 4.01409959 | 0.026603455 |
| *k__Bacteria\|p__Firmicutes\|c__Clostridia\|o__Lachnospirales\|f__Lachnospiraceae\|g__Lachnoclostridium* | 3.873230042 | CG | 3.250089476 | 0.033006258 |
| *k__Bacteria\|p__Firmicutes\|c__Clostridia\|o__Lachnospirales\|f__Lachnospiraceae\|g__Lachnospira* | 3.63988324 | CG | 3.234827379 | 0.04506077 |
| *k__Bacteria\|p__Bacteroidota\|c__Bacteroidia\|o__Bacteroidales\|f__Prevotellaceae\|g__Alloprevotella* | 3.296200022 | CG | 3.168482479 | 0.027949266 |
| *k__Bacteria\|p__Bacteroidota\|c__Bacteroidia\|o__Bacteroidales\|f__Tannerellaceae\|g__Parabacteroides* | 3.489001897 | CG | 3.102904087 | 0.000225599 |
| *k__Bacteria\|p__Firmicutes\|c__Clostridia\|o__Oscillospirales\|f__Oscillospiraceae\|g__Flavonifractor* | 2.522232063 | CG | 2.035288282 | 0.001280735 |
| *k__Bacteria\|p__Bacteroidota\|c__Bacteroidia\|o__Bacteroidales\|f__Tannerellaceae* | 3.489001897 | CG | 3.102904087 | 0.000225599 |
| *k__Bacteria\|p__Proteobacteria\|c__Gammaproteobacteria\|o__Burkholderiales\|f__Sutterellaceae* | 3.479925382 | CG | 3.047286843 | 0.032985993 |
| *k__Bacteria\|p__Proteobacteria\|c__Gammaproteobacteria\|o__Burkholderiales* | 3.481044332 | CG | 3.046675131 | 0.029651842 |

Table S3. Lefse analysis results based on KEGG level3 predicted pathways.

| **Biomarker names** | **Log value** | **Group** | **LDA score** | ***p* value** |
| --- | --- | --- | --- | --- |
| Enzymes with EC numbers | 4.367894306 | CG | 2.840726361 | 0.000314454 |
| Other glycan degradation [PATH:ko00511] | 3.529547312 | CG | 2.831834763 | 0.001026027 |
| Lysosome [PATH:ko04142] | 3.273338582 | CG | 2.690581921 | 0.000401445 |
| Amino sugar and nucleotide sugar metabolism [PATH:ko00520] | 4.106491968 | CG | 2.582385551 | 0.0082008 |
| O-Antigen nucleotide sugar biosynthesis [PATH:ko00541] | 3.736904974 | CG | 2.519792826 | 0.003490781 |
| Exosome [BR:ko04147] | 4.138472491 | CG | 2.465449172 | 0.015078498 |
| Antimicrobial resistance genes [BR:ko01504] | 3.480800903 | CG | 2.438834153 | 0.000207511 |
| General function prediction only | 3.690674813 | CG | 2.385414079 | 0.006776163 |
| Citrate cycle (TCA cycle) [PATH:ko00020] | 3.724434227 | CG | 2.36544062 | 0.001724112 |
| Glycosaminoglycan degradation [PATH:ko00531] | 3.044794407 | CG | 2.350371293 | 0.000401445 |
| Sphingolipid metabolism [PATH:ko00600] | 3.347529136 | CG | 2.344478852 | 0.005223309 |
| Cationic antimicrobial peptide (CAMP) resistance [PATH:ko01503] | 3.500069456 | CG | 2.33509333 | 0.000175197 |
| Structural proteins | 3.358055937 | CG | 2.322063756 | 0.006353264 |
| Carbon fixation pathways in prokaryotes [PATH:ko00720] | 3.92724626 | CG | 2.309514587 | 0.002466264 |
| Chaperones and folding catalysts [BR:ko03110] | 4.013972083 | CG | 2.308724018 | 0.023825745 |
| Biotin metabolism [PATH:ko00780] | 3.588458062 | CG | 2.284535935 | 9.56E-05 |
| Glycosphingolipid biosynthesis - ganglio series [PATH:ko00604] | 2.84229809 | CG | 2.283113502 | 0.000245363 |
| Various types of N-glycan biosynthesis [PATH:ko00513] | 2.842715244 | CG | 2.28310059 | 0.000266631 |
| Transport | 3.7067116 | CG | 2.266840915 | 0.04982695 |
| Pentose and glucuronate interconversions [PATH:ko00040] | 3.612528089 | CG | 2.247237102 | 0.017955818 |
| Glycosphingolipid biosynthesis - globo and isoglobo series [PATH:ko00603] | 3.126774048 | CG | 2.237627431 | 0.003999392 |
| Biofilm formation - Vibrio cholerae [PATH:ko05111] | 3.452220724 | CG | 2.230908839 | 0.000370217 |
| Glyoxylate and dicarboxylate metabolism [PATH:ko00630] | 3.854669953 | CG | 2.227760903 | 0.042825609 |
| Glycosyltransferases [BR:ko01003] | 3.445618098 | CG | 2.110974526 | 0.017955818 |
| Phenylalanine metabolism [PATH:ko00360] | 3.330054644 | CG | 2.026247198 | 0.000552641 |
| beta-Lactam resistance [PATH:ko01501] | 3.634909967 | CG | 2.008266276 | 0.007698627 |
| Ubiquinone and other terpenoid-quinone biosynthesis [PATH:ko00130] | 3.061877216 | CG | 2.004890254 | 0.038635609 |
| ABC transporters [PATH:ko02010] | 4.385578338 | RG | 2.91047299 | 0.026603455 |
| Transcription factors [BR:ko03000] | 4.205696908 | RG | 2.830287666 | 0.007698627 |
| Cell growth | 3.898461412 | RG | 2.712697896 | 0.020131508 |
| Ribosome biogenesis [BR:ko03009] | 4.260238896 | RG | 2.625483954 | 0.002645958 |
| Ribosome [PATH:ko03010] | 4.330397431 | RG | 2.537574081 | 0.012615307 |
| Ribosome [BR:ko03011] | 4.330397431 | RG | 2.537574081 | 0.012615307 |
| Quorum sensing [PATH:ko02024] | 4.112830084 | RG | 2.497327274 | 0.019016474 |
| Transfer RNA biogenesis [BR:ko03016] | 4.374445855 | RG | 2.46859236 | 0.009887797 |
| Starch and sucrose metabolism [PATH:ko00500] | 4.076523446 | RG | 2.459042663 | 0.023825745 |
| DNA repair and recombination proteins [BR:ko03400] | 4.438599117 | RG | 2.444727366 | 0.00304172 |
| Peptidoglycan biosynthesis and degradation proteins [BR:ko01011] | 4.047239694 | RG | 2.443146467 | 0.000552641 |
| Peptidoglycan biosynthesis [PATH:ko00550] | 3.93485394 | RG | 2.440233366 | 0.000435121 |
| Aminoacyl-tRNA biosynthesis [PATH:ko00970] | 4.004762954 | RG | 2.345755596 | 0.001853652 |
| Transcription machinery [BR:ko03021] | 3.970044904 | RG | 2.307478936 | 0.015078498 |
| Mitochondrial biogenesis [BR:ko03029] | 4.06515586 | RG | 2.216581382 | 0.006776163 |
| Glycerolipid metabolism [PATH:ko00561] | 3.551588989 | RG | 2.18003818 | 0.004889127 |
| Cysteine and methionine metabolism [PATH:ko00270] | 4.019200168 | RG | 2.160901051 | 0.003490781 |
| Pantothenate and CoA biosynthesis [PATH:ko00770] | 3.769890579 | RG | 2.147117305 | 8.75E-05 |
| Cytoskeleton proteins [BR:ko04812] | 3.635467252 | RG | 2.129689961 | 0.009887797 |
| Chromosome and associated proteins [BR:ko03036] | 4.204406241 | RG | 2.078595877 | 0.04982695 |
